# JMJD8 Promotes Malignant Progression of Lung Cancer by Maintaining EGFR Stability and EGFR/PI3K/AKT Pathway Activation

**DOI:** 10.7150/jca.50234

**Published:** 2021-01-01

**Authors:** Bo Zhang, Yao Zhang, Xizi Jiang, Hongbo Su, Qiongzi Wang, Muli Wudu, Jun Jiang, Hongjiu Ren, Yitong Xu, Zongang Liu, Xueshan Qiu

**Affiliations:** 1 Department of Pathology, First Affiliated Hospital and College of Basic Medical Sciences, China Medical University, Shenyang, China.; 2 Department of Thoracic Surgical, Shengjing Hospital Affiliated with China Medical University, Shenyang, China.

**Keywords:** cell invasion, cell proliferation, epidermal growth factor receptor, JMJD8, PI3K/AKT

## Abstract

JMJD8 is a JmjC domain-containing protein that has not been widely examined, despite its potential role in malignant tumor development. The underlying biological functions and molecular mechanisms of JMJD8 in non-small-cell lung cancer (NSCLC) remain unclear. Herein, we explored the relationship between JMJD8 and the activation of malignancy pathways in NSCLC. Immunohistochemical analyses revealed that high JMJD8 expression significantly correlated with cell differentiation and advanced TNM stages of NSCLC. The overexpression of JMJD8 promoted cell proliferation and invasion* in vitro*. Upon JMJD8 knockdown in lung cancer cell lines, cyclin B1, RhoA, RhoC, MMP9, and N-cadherin were down-regulated, and p21 and E-cadherin were conversely up-regulated. Key factors in the PI3K/AKT signaling pathway, such as p‑AKT, showed clear decreases in expression; additionally, the expression of epidermal growth factor receptor (EGFR), which functions upstream of PI3K, was altered. Co-immunoprecipitation experiments indicated that JMJD8 interacts with EGFR, and JMJD8 knockdown accelerated EGFR degradation. Our results suggested that JMJD8 functions as an oncogenic regulator in NSCLC. We found that JMJD8 promotes carcinogenic activity in NSCLC cells by facilitating EGFR stability, thereby activating the downstream PI3K/AKT signaling pathway. JMJD8 shows potential as a prognostic marker for lung cancer patients, providing a new target for therapeutic strategies.

## Introduction

Lung cancer is the fastest-growing malignant tumor disease, displaying both high morbidity and mortality; therefore, it poses a major threat to human health globally 1-3. There are two broad histological subtypes of lung cancer: non-small-cell lung cancer NSCLC (85% of cases) and small-cell lung cancer (SCLC) (15% of cases). NSCLC mainly includes lung squamous cell carcinoma and lung adenocarcinoma. The current treatment of lung cancer mainly includes surgery, radiotherapy, and chemotherapy, but the long-term survival of lung cancer is still unsatisfactory, and the five-year survival rate has been maintained at 15% 4. In addition, immunotherapy and targeted therapies with monoclonal antibodies and tyrosine kinase inhibitors have also been limited their effective treatment of lung cancer due to the acquired resistance. Therefore, it is significant to understand the mechanisms involved in the progression of NSCLC 5.

Epidermal growth factor receptor (EGFR) is a tyrosine kinase receptor that is often highly expressed or overactivated in NSCLC. EGFR can regulate the cycle of lung cancer cells through signal transduction pathways, promote tumor cell proliferation, induce angiogenesis, and promote tumor spread and metastasis. Accordingly, EGFR can reduce the killing effect of cytotoxic drugs on tumor cells 6, 7. The EGFR protein is divided into three domains: the extracellular ligand-binding domain, the transmembrane domain, and the intracellular kinase domain. The EGFR protein participates in various signaling pathways, and the phosphorylation of tyrosine residues in its intracellular kinase domain can initiate downstream signaling pathways 8 including MAPK/ERK and PI3K/AKT 9. On the cell surface, EGFR binds to a target ligand and is internalized into the cell via endocytosis. After late endosome signaling, EGFR is typically directed to the lysosomes for degradation, or sent back to the cell surface for reuse.

The PI3K/AKT pathway is frequently activated in various human cancers; it can lead to the malignant transformation of cells, and is associated with apoptosis and tumor angiogenesis. PI3Ks can be divided into three categories; the IA class is the most extensive, and includes the p85 regulatory subunit and the p110 catalytic subunit 10, 11. AKT serves as a hub protein in the signaling pathway. It functions by phosphorylating downstream target proteins for multiple effects. AKT stimulates cell invasiveness by increasing the production of matrix metalloproteinase 9 (MMP9) and regulating the expression of proteins involved in epithelial mesenchymal transition (EMT) 12, 13 Previous studies have shown that PI3K/AKT overactivation can exacerbate the symptoms of glioma, colon cancer, and ovarian cancer 14.

Jumonji C domain-containing (JMJD) proteins are classified into seven distinct subgroups based on their demethylation activity and domain architecture 15. They are known to function in cell apoptosis, proliferation, and stress response. Recent studies have shown that many JMJD proteins are closely related to the development and progression of cancer. In oral squamous cell carcinoma, JMJD5 was found to induce apoptosis by influencing p53/nuclear factor (NF)-κB signaling 16. In gastric cancer, high JMJD3 expression is associated with a reduced overall survival rate 17.

JMJD8 belongs to the JMJD superfamily, but is evolutionarily distant from the other members and has a relatively low molecular weight 18. This protein has not been thoroughly studied; thus, its mechanisms and functions remain unclear. A previous study showed that the JmjC barrel protein domain possesses histone demethylase catalytic activity 15. However, the demethylation function of JMJD8 has not been clarified. JMJD8 was reported to promote tumor necrosis factor (TNF)-induced NF-κB signaling and apoptosis in H293T cells 19. Furthermore, the knockdown of JMJD8 strongly inhibited squamous cell carcinoma cell invasion 20 and affected DU145 cell viability 21. Based on these results, we predicted that JMJD8 would affect the occurrence and development of NSCLC.

To evaluate the underlying functions and mechanisms of JMJD8 in non-small-cell lung cancer (NSCLC), in this study, we analyzed the role of JMJD8 in promoting proliferation and invasion of lung cancer cells, as well as its relationship with the EGFR pathway.

## Materials and Methods

### Patients and specimens

Tissue samples were collected from 169 patients diagnosed with NSCLC who had undergone complete resection at the First Affiliated Hospital of China Medical University between 2010 and 2017. Case information was derived from hospital records, and patient consent, with signature, was obtained. Patients were not administered chemotherapy or radiotherapy before undergoing surgery, and received regular treatment after surgery. This study was conducted according to the Declaration of Helsinki, and approved by the Medical Research Ethics Committee of China Medical University.

### Immunohistochemistry

Immunohistochemical reagents were purchased from Maxim (KIT-9922; Fuzhou, China). JMJD8 rabbit polyclonal antibodies were purchased from Abnova (1:500; Taipei, Taiwan). Paraffinized tissue sections were placed in xylene and gradient alcohol for dewaxing, transferred to a citrate buffer (0.01M, pH=6.0) for antigen retrieval (endogenous peroxidase activity was blocked by hydrogen peroxide), and incubated in normal goat serum for 30 min. The antibodies were incubated overnight at 4 °C, and then DAB (DAB-0031; Fuzhou, China) substrate solution was applied for antibody staining for 3min. Nuclei were stained with hematoxylin. Two investigators blinded to the clinical data semi-quantitatively scored the slides by evaluating the staining intensity and percentage of stained cells in representative areas.

Staining intensity was scored as follows: 0, no staining; 1, weak staining (light yellow); 2, medium staining (khaki); and 3, strong staining (brown). The proportion of positive cells was scored as follows: 1, 1-25%; 2, 26-50%; 3, 51-75%; and 4, 76-100%. A final score of 0-12 was obtained by multiplying the staining intensity and the proportion of positively stained cells. The cut-off value was set at 6, tumor samples with scores ≥6 were recognized as positive expression, scores between 1 and 6 were categorized as weak expression, and a score of 0 was considered as negative. (For example, if the staining intensity is 3, proportion of positive cells is 77%, the final score of JMJD8 expression is 3*4=12).

### Cell culture

All cell lines were purchased from the Cell Bank of the China Academy of Sciences (Shanghai, China) and maintained in a 5% CO_2_ incubator at 37 °C. A549, H1299, H460, H661, and H292 cells were cultured with RPMI 1640 medium (Gibco, Grand Island, NY, USA). SK-MES-1 cells were cultured in minimal essential medium (Gibco), and HBE cells were cultured in DMEM (Gibco). All media were supplemented with 10% fetal bovine serum (FBS) (FB15015; Clark Biosciences, Richmond, VA, USA).

### Transfection and interference

pCMV6-JMJD8 plasmids were purchased from Taihe Biotechnology (Beijing, China), and pCMV6 empty vector was purchased from Origene (Rockville, MD, USA). Stable clonal cell lines were selected with G418 (Thermo Fisher Scientific, Waltham, MA, USA). Short interfering RNA (siRNA) targeting JMJD8 and negative control siRNA (NC) were purchased from Ruibo (Guangzhou, China). The siRNA (50 nM) used for the specific targeting of JMJD8 had the following sequence: 5′-GGTACTCAGAAGTGATCTA-3′, according to the instructions, cells were transiently transfected by Lipofectamine 3000 (Invitrogen, Carlsbad, CA, USA).

The cells were stimulated with 100 ng/mL EGF (PeproTech, Rocky Hill, NJ, USA) for the indicated time periods after 16 hours of serum starvation, and 1 hour of incubation with cycloheximide (CHX, MedChemExpress, NJ, USA).

### Immunofluorescence

Cells were seeded in 24-well plates, fixed in pre-cooled methanol for 10 min, and then permeabilized with 1% Triton X-100 for 15 min, after washing the suspended cells with PBS. Next, the cells were blocked with goat serum for 2 h and incubated with JMJD8 antibodies (1:5, Santa Cruz Biotechnology, Dallas, TX, USA) overnight at 4 °C. Subsequently, samples were incubated with the corresponding HRP-conjugated secondary antibody solution for 2 h. Nuclei were stained with DAPI. Cell images were obtained using an Olympus FV1000 laser scanning confocal microscope (Olympus, Tokyo, Japan).

### Western blotting

Cells and tissues were lysed in a lysis buffer (P0013F; Beyotime Biosciences, Shanghai, China) and total protein (50 µg) was separated by 8% or 10% SDS-PAGE and transferred to polyvinylidene fluoride membranes (Millipore, Billerica, MA, USA). Next, the membrane was blocked with 5% skim milk for 2 h at room temperature to reduce non-specific binding. Subsequently, the membranes were incubated with primary antibody overnight at 4 °C. Following incubation with HRP-conjugated anti-rabbit/-mouse IgG at 37 °C for 2 h, the bands were quantified using ImageJ software (NIH, Bethesda, MD, USA). Cyclin B1 (#4138, 1:1000), CyclinA2 (mAb#91500, 1:1000), CyclinD1 (mAb#55506, 1:1000), CDK1 (mAb#9116, 1:1000),RhoA (mAb#2117, 1:1000), RhoC (mAb#3430, 1:1000), PI3K (mAb#13666, 1:1000), AKT (mAb#4685, 1:1000), p-Akt (ser473) (#4060, 1:1000), EGFR (mAb#4267, 1:1000), p-EGFR (Tyr1068) (mAb#3777, 1:1000) and p-ERK (mAb#4370, 1:1000) antibodies were purchased from Cell Signaling Technology (Danvers, MA, USA). Antibodies for the following were purchased from Proteintech (Rosemont, IL, USA): p21 (10355-1-AP, 1:500), MMP9 (10375-2-AP, 1:500), MMP7 (10374-2-AP, 1:500), N-cadherin (22018-1-AP, 1:500), vimentin (10366-1-AP, 1:500), Snail (10399-1-AP, 1:500), Slug (12129-1-AP, 1:500), E-cadherin (20874-1-AP, 1:500) and RAS (12063-1-AP, 1:500). JMJD8 (sc-515520, 1:150) antibodies was purchased from Santa Cruz Biotechnology (Dallas, TX, USA). Protein bands were visualized with ECL (Share-bio Biotechnology Co., Shanghai, China) and detected using a bio-imaging system (DNR, Neve Yamin, Israel). The bands were quantified using ImageJ software. The relative protein levels were calculated using β-actin or GAPDH as an internal control.

### Cell proliferation and colony formation assays

An MTT assay was used to detect the proliferative capacity of cells over 5 consecutive days. Cells were briefly cultured in 96-well plates at a starting concentration of 3 × 10^3^ cells/well. Cells were incubated at 37 °C for 4 h in MTT solution; subsequently, the media were aspirated from the cultures, and 100 µL/well DMSO was added. The optical density was measured at 490 nm using a spectrophotometer.

For the colony formation assay, cells were cultured in 6-wells plates at a density of 1 × 10^3^ cells/well for 11 days. The cells were then fixed with pre-cooled methanol and stained with crystal violet solution. Colonies with more than 50 cells were counted. Each experiment was performed at least three times independently.

### Cell invasion analysis

Cell invasion assays were performed using 24-well Transwell chambers containing inserts with a pore size of 8 μm (Costar, Inc., Corning, NY, USA); these inserts were coated with 100 μL Matrigel (1:7; BD Biosciences, Franklin Lakes, NJ, USA). Approximately 3 × 10^4^ A549 cells or 6 × 10^4^ H1299 cells were placed into the upper Transwell chamber with 2% FBS, whereas the lower chamber contained 20% FBS. After incubation at 37 °C for 24 h, the cells were fixed with pre-chilled methanol, and then stained with crystal violet for 10 min.

### Co-immunoprecipitation (co-IP)

Cells were cultured in 10-cm diameter plates and then lysed in a lysis buffer (P0013F; Beyotime Biosciences, Shanghai, China) to obtain total protein. Following this, 10 μg anti-JMJD8 (sc-515520, Santa Cruz Biotechnology, USA) or anti-IgG antibodies (dependent upon the experimental condition) was added to the supernatant contains 200μg of protein, and the samples were incubated overnight at 4 °C with shaking. The immunocomplex was captured by adding 25 μL of protein A/G agarose beads, and the supernatant was removed. The solution was then gently mixed with 2× loading buffer after washing three times with RIPA buffer. The sample was boiled for 10 min. Interaction was then evaluated by electrophoresis.

### Quantitative real-time PCR

Assays were performed as described previously 22. Primer sequences were as follows: *JMJD8* forward, 5′-CTAGCCGAGGAAGGTGGAGATGAAGA-3′; *JMJD8* reverse, 5′-TTTACCCACCTGAGAAGACGCCAGA-3′; *GAPDH* forward, 5′-GGAGCGAGATCCCTCCAAAAT-3′; *GAPDH* reverse, 5′-GGCTGTTGTCATACTTCTCATGG-3′; *EGFR* forward: 5′-GGAGAACTGCCAGAAACTGACC-3′; *EGFR* reverse: 5′-GCCTACAGCACACTGGTTG-3′. The fold change of the gene expression was calculated by the 2^-△△C^ method and relative transcript levels of genes were normalized to GAPDH mRNA levels.

### Bioinformatics analysis

We used the GEPIA database (http://gepia.cancer-pku.cn/), a public online database, it was used to investigate the correlation between JMJD8 and overall survival.

### Statistical analysis

SPSS version 22.0 (SPSS, Chicago, IL, USA) was used for all statistical analyses. Correlations between JMJD8 expression and clinicopathological factors were determined by the Chi-square test. All other data were compared by a paired Student's *t*-test. A value of *P* < 0.05 was considered to be statistically significant.

## Results

### JMJD8 overexpression is associated with NSCLC progression

**Figure 1:**
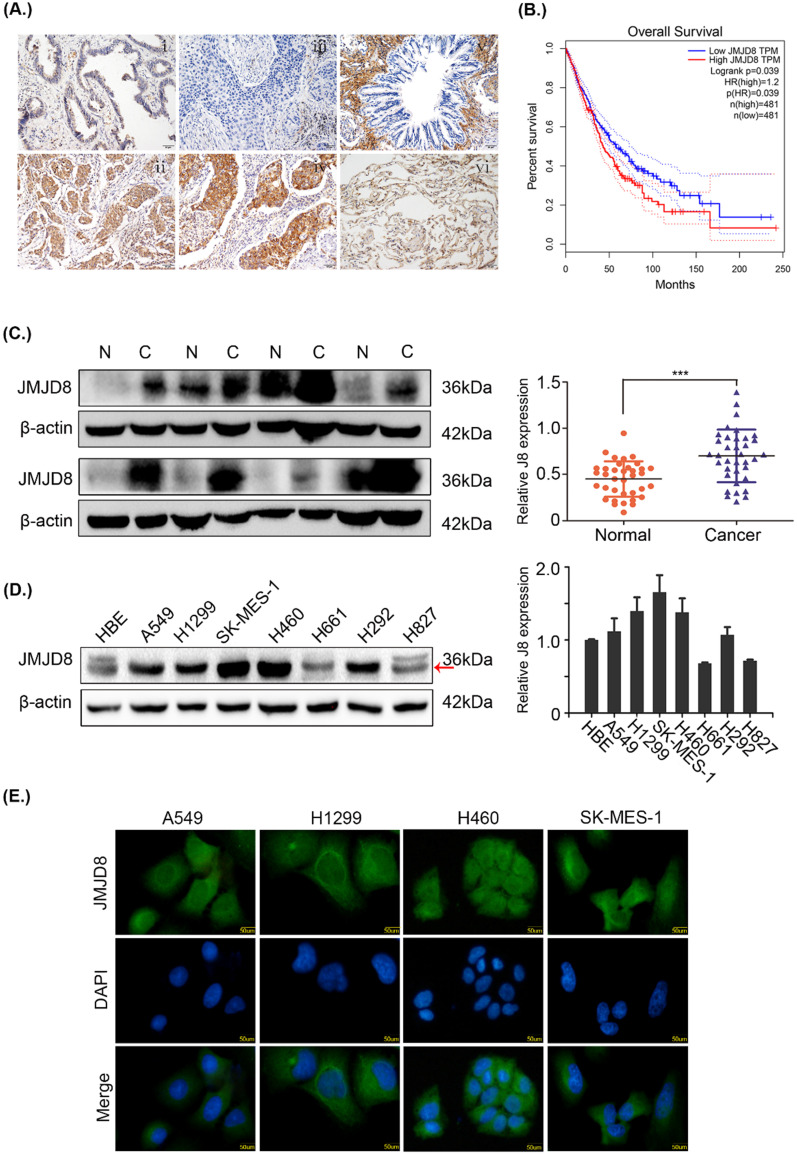
**JMJD8 overexpression is associated with NSCLC progression. (A)** The expression of JMJD8 as determined by immunohistochemistry. (i) Well-differentiated adenocarcinoma. (ii) Poorly differentiated adenocarcinoma. (iii) Well-differentiated squamous cell carcinoma. (iv) Poorly differentiated squamous cell carcinoma. (v) Normal bronchial epithelium. (vi) Alveolus. Negative or weak positive expression was observed in (I, iii, v, vi). Magnification, × 200. **(B)** Patients with low JMJD8 expression had high overall survival in lung adenocarcinoma (LUAD) and lung square cell carcinoma (LUSC) based on GEPIA. **(C)** Expression of JMJD8 in 36 paired tumors (C) and adjacent noncancerous tissues (N); western blotting shows partial representative samples. β-actin was used as a loading control. The results were quantified using ImageJ, and intensity values were normalized to the β-actin band. **(D)** Immunoblots showing the expression of JMJD8 in lung cancer cells and in HBE (normal bronchial epithelial) cells. **(E)** Immunofluorescence assay to detect the localization of JMJD8 expression in NSCLC cell lines. *P < 0.05; **P < 0.01 (Data are presented as the mean ± SD of three independent experiments).

The expression of JMJD8 was measured by immunohistochemistry in 169 samples. In poorly differentiated adenocarcinomas and squamous cell carcinomas, JMJD8 showed strong staining and weak staining, respectively, in the bronchial epithelium (Fig. 1A). Based on the GEPIA database, JMJD8 was negatively associated with overall survival and disease-free survival of lung adenocarcinoma and squamous cell carcinoma (Fig. 1B). We then analyzed the relationship between JMJD8 expression and clinicopathological parameters. The expression of JMJD8 was confirmed to be associated with poor differentiation (*P* < 0.01) and advanced TNM stages (*P* = 0.026) of NSCLC (Table 1). Notably, JMJD8 was more highly expressed in tumor tissues than in adjacent tissues (Fig. 1C). Immunoblotting showed that JMJD8 was expressed at higher levels in lung cancer cells than in HBE (normal bronchial epithelial) cells (Fig. 1D). Immunohistochemical analysis showed that the expression of JMJD8 in lung adenocarcinoma has statistically significant; and thus, we selected A549 lung adenocarcinoma and H1299 (moderate JMJD8 expression) cells to perform subsequent experiments. Furthermore, immunofluorescence to detect JMJD8 expression in A549, H1299, 460, and SK-MES-1 cells suggested that JMJD8 was mainly expressed in the cytoplasm (Fig. 1E).

### JMJD8 promotes NSCLC cell proliferation

**Figure 2:**
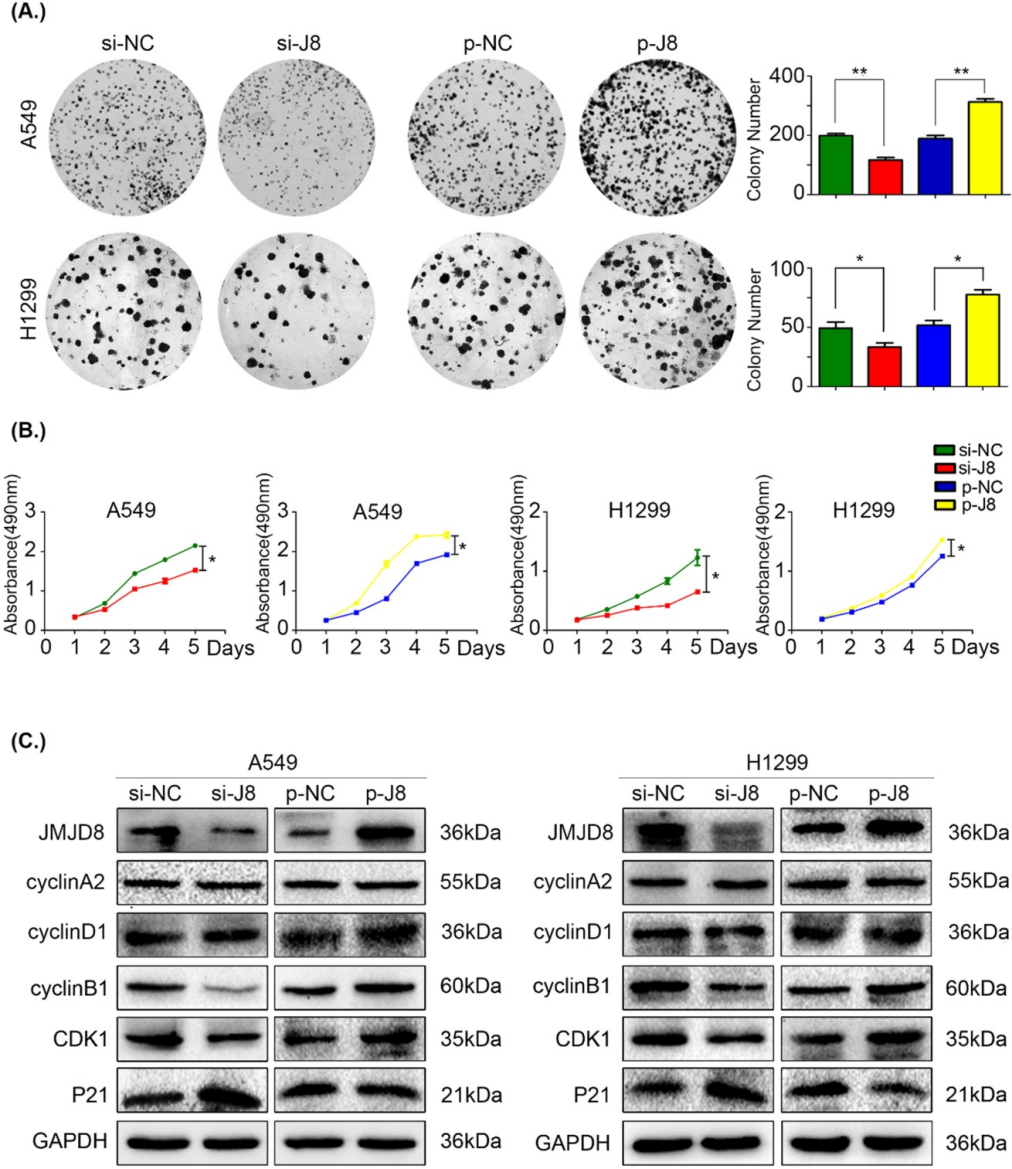
**JMJD8 promotes the proliferation of NSCLC cells. (A, B)** H1299 cells overexpressing JMJD8 or with JMJD8 knockdown via siRNA were subjected to **(A)** colony formation assay and **(B)** MTT assay. Increased proliferation was observed after JMJD8 overexpression, and decreased proliferation was observed after JMJD8 knockdown in tumor cells. **(C)** Changes in the expression of cell cycle progression-related proteins in A549 and H1299 cells. *P < 0.05; **P < 0.01 (Data are presented as the mean ± SD of three independent experiments).

Based on the results of immunohistochemistry, we evaluated cell proliferation using an MTT assay and colony formation assay. JMJD8 expression was knocked down in A549 and H1299 cells using siRNA, and the control group formed larger and more abundant colonies (Fig. 2A). Similarly, the growth of the si-JMJD8 cells was obviously lower than that of the negative control group (Fig. 2B). We next detected changes in the expression of cell cycle progression-related proteins 23 and found that cyclin B1 and CDK1 were down-regulated when JMJD8 was knocked down. The expression of p21 (a cyclin-dependent kinase inhibitor) increased with JMJD8 levels (Fig. 2C). In addition, we stably overexpressed JMJD8 using an appropriate plasmid, which resulted in the opposite effects (Fig. 2C). No apparent changes in other proteins (e.g., cyclin A2 and cyclin D1) were observed after upregulation or down-regulation of JMJD8.

### JMJD8 promotes the invasion of NSCLC cells

**Figure 3:**
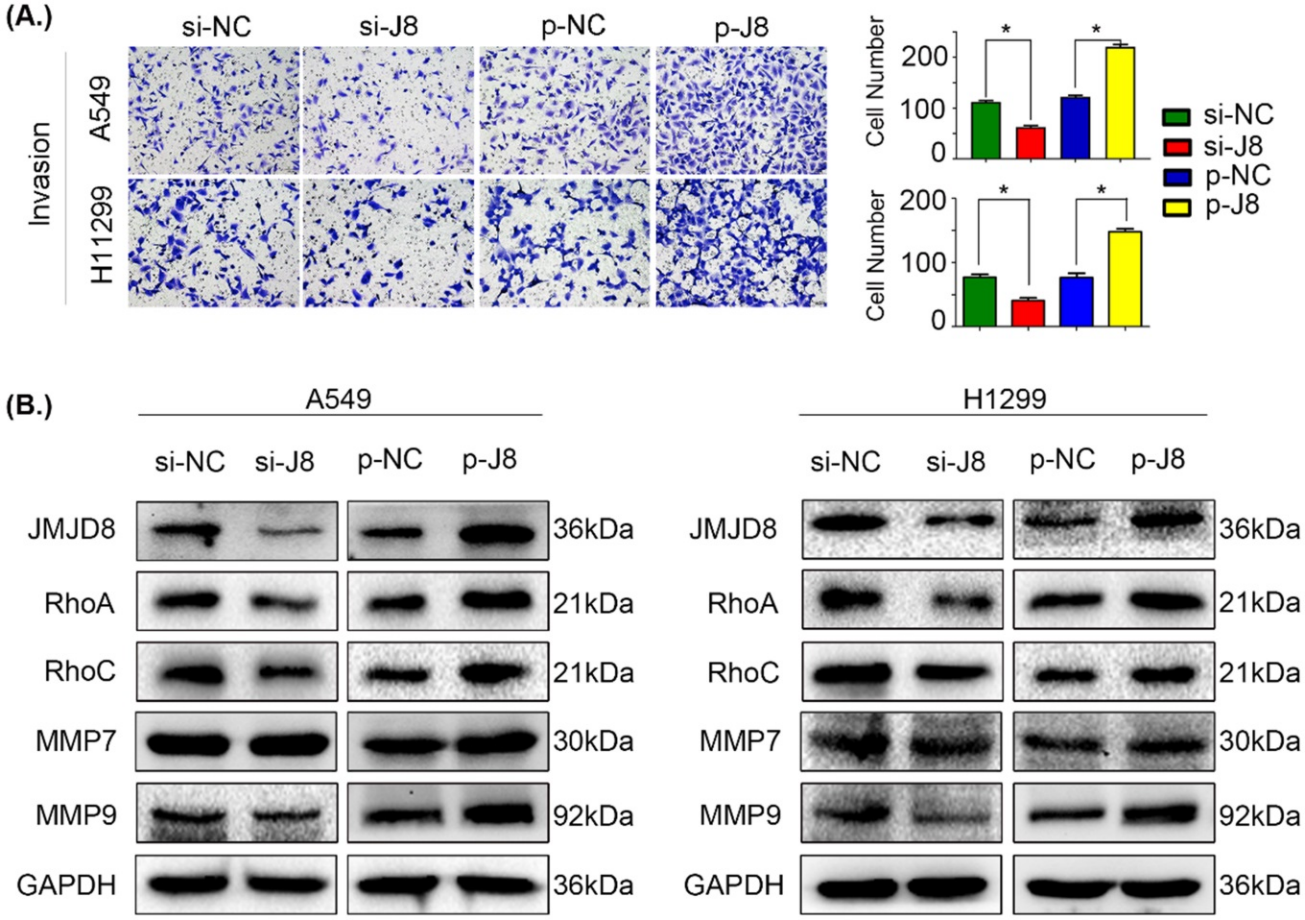
** JMJD8 promotes invasion in NSCLC. (A)** JMJD8 overexpression in cells increased the invasive potential of cells, and JMJD8 knockdown reduced the invasive capacity of cells accordingly.** (B)** Effects of JMJD8 levels on the expression of proteins related to cell invasion in A549 and H1299 cells. Western blotting results showing increased expression of proteins related to cell invasion after JMJD8 overexpression. *P < 0.05; **P < 0.01 (Data are presented as the mean ± SD of three independent experiments).

We next evaluated the influence of JMJD8 on the invasive potential of NSCLC cells by genetically manipulating the expression of JMJD8. Overexpression of JMJD8 enhanced the invasive abilities of A549 and H1299 cells compared with those of the control group (Fig. 3A). Variations in JMJD8 expression influenced the expression of proteins related to cell invasion 24, including RhoA, RhoC, and MMP9, consistent with the invasion assay (Fig. 3B). When JMJD8 was knocked down, opposite results were observed. There were no apparent changes in expression of other proteins such as MMP7 (Fig. 3B).

### JMJD8 positively regulates the expression of EMT-related proteins

**Figure 4:**
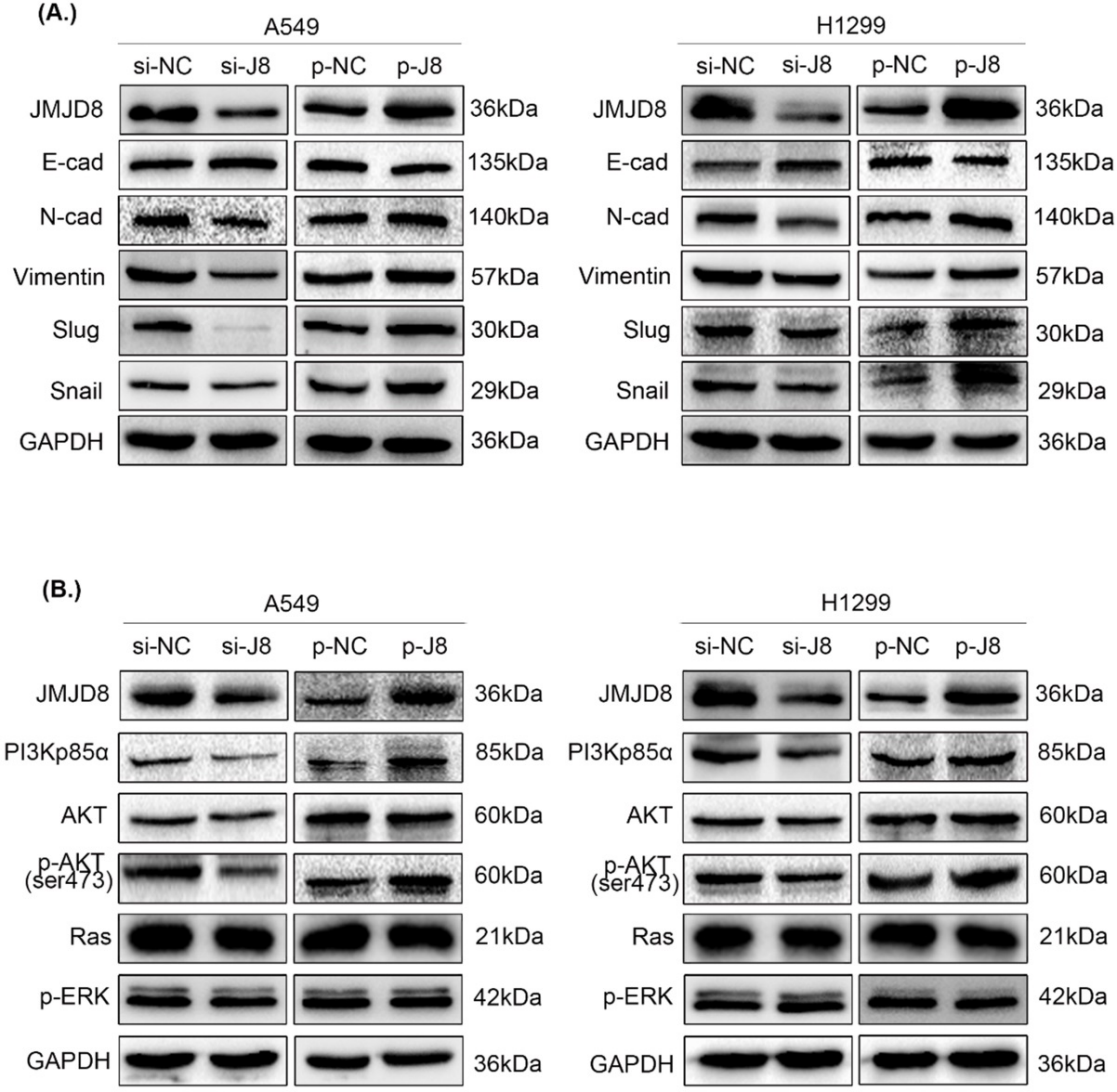
**Effect of JMJD8 on the expression of EMT-related proteins and key molecules in the PI3K/AKT signaling pathway. (A)** Western blotting results showing the expression levels of EMT-related proteins in A549 and H1299 cells.** (B)** JMJD8 overexpression in A549 and H1299 cells resulted in the upregulation of phosphorylated AKT and PI3Kp85α levels. Conversely, JMJD8 knockdown decreased the level of phosphorylated AKT and PI3Kp85α levels, however, the expression of key molecules in the RAS/MAPK/ERK signaling pathway showed no significant changes.

Subsequently, we examined changes in the expression of proteins involved in EMT. Slug, Snail, N-cadherin, and vimentin showed increased expression, while E-cadherin showed decreased expression after up-regulation of JMJD8; conversely, the knockdown of JMJD8 expression by RNA interference showed the opposite results (Fig. 4A).

### JMJD8 promotes NSCLC proliferation and invasion via PI3K/AKT signaling

To evaluate the specific biological mechanisms associated with the effects of JMJD8, we analyzed several vital signaling pathways, including PI3K/AKT and RAS/MAPK/ERK that are involved in regulating NSCLC proliferation and invasion. The expression of key factors in the PI3K/AKT signaling pathway was shown to be affected by JMJD8 expression. The protein levels of PI3Kp85α and p-AKT were attenuated after RNA interference of JMJD8 in A549 cells, and were enhanced after overexpression of JMJD8; however, the expression of AKT, RAS, and p-ERK showed no significant changes (Fig. 4B). Similar results were obtained in H1299 cells.

### JMJD8 interacts with EGFR and regulates its expression

**Figure 5:**
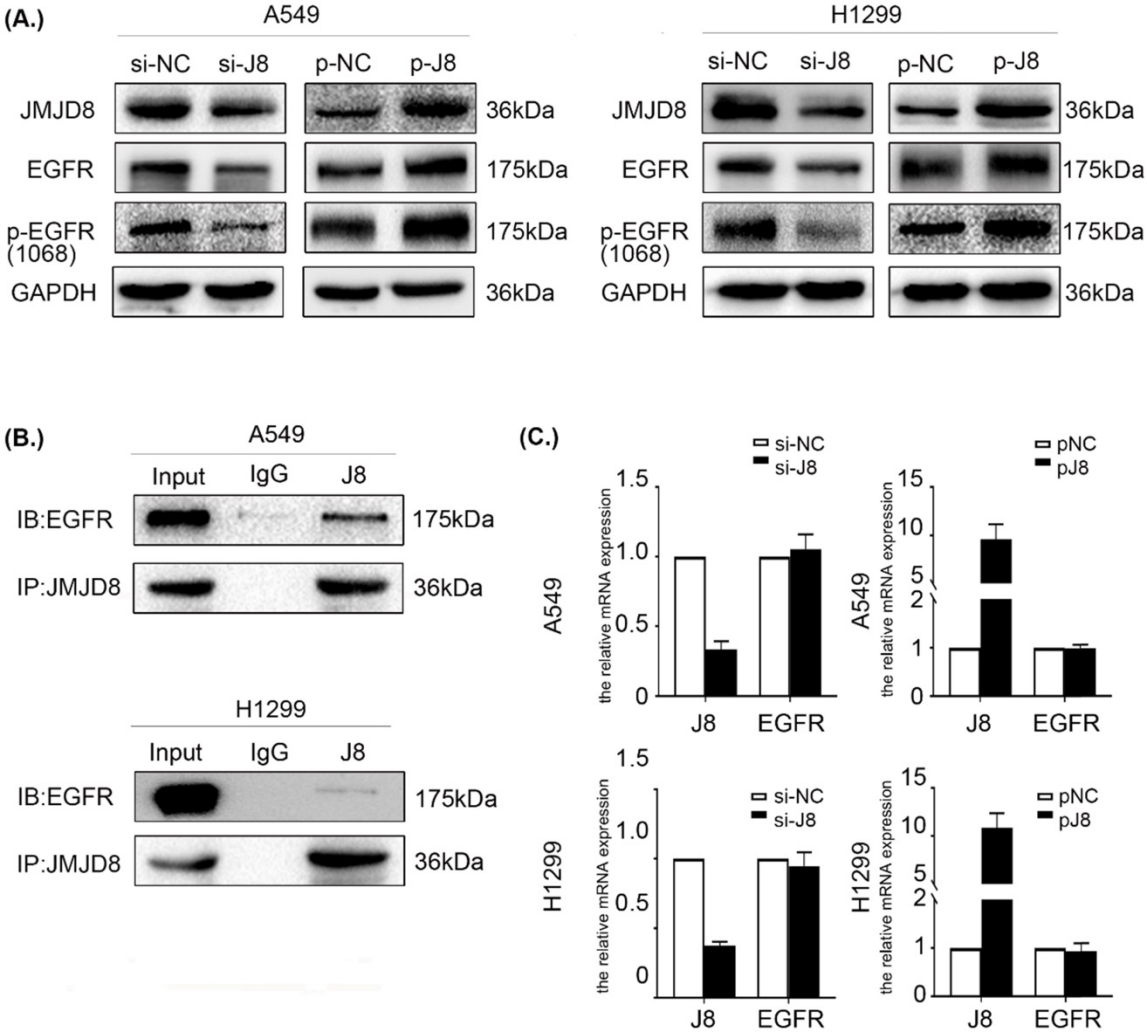
** JMJD8 interacts with EGFR and regulates its expression. (A)** EGFR level changes with that of JMJD8; depletion of JMJD8 significantly reduced EGFR and p-EGFR levels. **(B)** Endogenous immunoprecipitation results showing that JMJD8 bound to EGFR in A549 and H1299 cells. **(C)** mRNA analysis of EGFR after JMJD8 knockdown or overexpression in A549 cells and H1299 cells.

The data described above showed that JMJD8 regulates the malignant behavior of NSCLC via PI3K/AKT signaling, which occurs downstream of EGFR. EGFR is a multifunctional membrane glycoprotein that is found in various tissues. We speculated that JMJD8 might modulate PI3K/AKT signaling via regulating EGFR. Thus, we examined the impact of JMJD8 on EGFR signaling in NSCLC cells. Interestingly, EGFR expression in the si-JMJD8 group was lower than that in the corresponding control group, and JMJD8 up-regulation increased the expression of both EGFR and p-EGFR (Fig. 5A). We then performed a co-IP assay, which showed that JMJD8 could bind to EGFR in A549 and H1299 cells (Fig. 5B). Finally, qPCR analysis was performed to assess *EGFR* mRNA synthesis following JMJD8 manipulation. The results revealed that JMJD8 did not interfere with *EGFR* mRNA levels, suggesting that JMJD8 may regulate EGFR expression at a posttranslational level (Fig. 5C).

### JMJD8 facilitates EGFR stability

**Figure 6:**
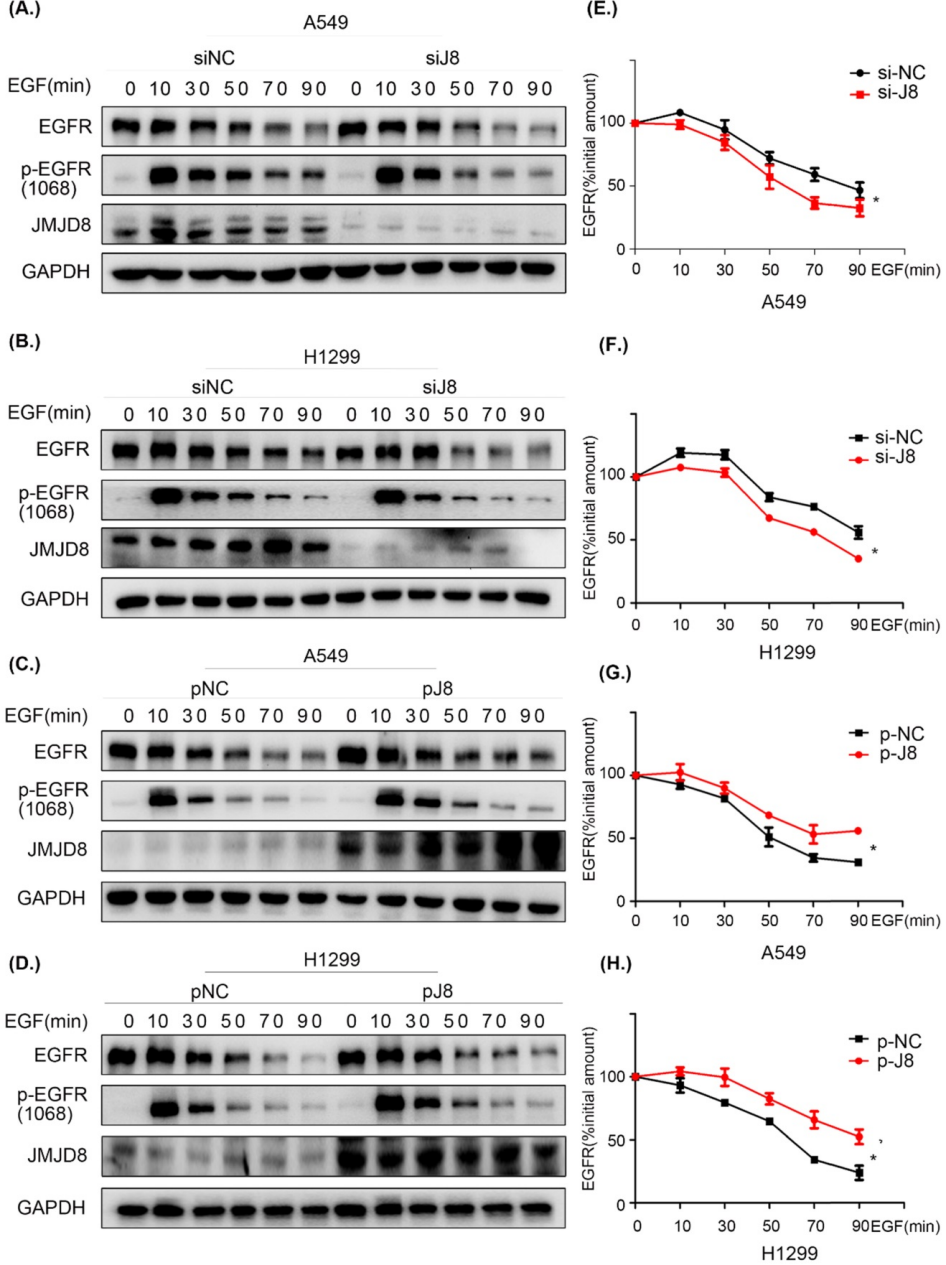
** JMJD8 regulates EGFR stability. (A, B)** EGF-stimulated cells were transfected with si-JMJD8 at the indicated times (0, 10, 30, 50, 70, 90 min) after treatment with cycloheximide (CHX; 150 ng/mL) for 1 h; the degradation of EGFR was significantly enhanced by JMJD8 silencing. **(C, D)** Regulation of EGFR in A549 cells and H1299 cells overexpressing JMJD8. **(E-H)** Relative quantification analysis of the results shown in **(A-D).** Western blot bands corresponding to EGFR was quantified and normalized against GAPDH. The data were normalized to A549(si-NC, p-NC) or H1299 (si-NC, p-NC) cells without EGF stimulation, which was considered as 100%. *P < 0.05, **P < 0.01 (Data are presented as the mean ± SD of three independent experiments).

To elucidate the mechanism through which EGFR is regulated by JMJD8, cells were serum-starved for 16 h and treated with cycloheximide (CHX) for 1 h, followed by stimulation with EGF for different time periods. The levels of EGFR were then determined by western blotting. JMJD8 down-regulation promoted EGFR degradation after EGF stimulation in both the A549 and H1299 cell lines (Fig. 6A, 6B). Additionally, the half-life (t_1/2_) of EGFR was significantly decreased after JMJD8 knockdown (Fig. 6E, 6F). Overexpressing JMJD8 in A549 and H1299 cells attenuated EGFR degradation after EGF stimulation (Fig. 6C, 6D, 6G, 6H). Thus, JMJD8 may modulate EGFR expression by suppressing its degradation.

## Discussion

In this study, we have identified a new connection between JMJD8 and EGFR, which provides a basis for further exploring the role of EGFR signaling in the proliferation, invasion and epithelial-mesenchymal transition of NSCLC cells promoted by JMJD8.

We investigated the relationship between JMJD8 and malignancy in NSCLC through which JMJD8 affects NSCLC malignancy using cultured A549 and H1299 cells, and found that JMJD8 has a major influence on the biological behavior of NSCLC, including on its proliferation and invasion, and affects the expression of functional proteins (cyclin B1, p21, MMP9) and key proteins (p-EGFR, p-AKT) in malignancy-related pathways. In addition, we found that JMJD8 interacts with EGFR to affect its stability. However, various aspects of the mechanism through which this occurred remain unclear. Whether the relationship between JMJD8 and EGFR is direct or indirect and how JMJD8 regulates EGFR degradation in cancer cells remains unknown.

Internalization of EGFR is thought to initiate the termination of the signaling from activated EGFR. EGFR internalization processes can be divided into ligand-dependent processes or non-ligand-dependent processes 25; ligand-mediated EGFR activation is achieved by conformational changes in the receptor's extracellular domain after ligand binding 26. There are two major destinations for EGFR in early endosomal cells: the cell surface (a return from the interior), or to the lysosome for degradation. Internalization and lysosomal-mediated EGFR degradation are important negative feedback mechanisms regulating EGFR signaling 27-29. Dysregulated EGFR endocytosis, whether due to delayed transportation to early endosomes or from early endosomes to late endosomes, affects the stability of the protein, enhances its signal transduction ability, and may lead to tumorigenesis. Another essential factor in the degradation of EGFR after internalization is ubiquitination. EGFR ubiquitination occurs via the Cbl domain of E3 ligase, and Cbl is recruited to activate EGFR via two different mechanisms: it can interact directly with the receptor at the pY1045 site, or indirectly by interacting with Grb2 at pY1068/pY1086 30, 31.

It remains unknown whether JMJD8 affects EGFR stability by disrupting the ubiquitination pathway of EGFR or by interrupting its endosomal transport. Future research needs to study in detail the mechanism of how JMJD precisely regulates the stability of EGFR.

A series of studies revealed EGFR overexpression or over-activation signaling pathway in NSCLC 32. EGFR overexpression leads not only to targeted EGFR drug resistance but also to tumor resistance to multiple chemotherapy drugs 33. In some cases, there is no direct correlation between gene copy number and EGFR protein expression, indicating that EGFR overexpression might be associated with dysregulated endocytosis and degradation. Enhancing the stability of EGFR will lead to the activation of downstream signaling pathways, cell transformation, and tumorigenesis. Therefore, studies on the effect of JMJD8 on the stability of EGFR and its downstream pathways might provide an experimental basis for new targeting molecules and will help to develop new treatment strategies.

In summary, our study demonstrates a new mechanism through which JMJD8 controls EGFR signaling in NSCLC cells. We revealed that JMJD8 plays a significant role in the proliferation, invasion and epithelial-mesenchymal transition of NSCLC cells by interacting with EGFR and enhancing its stability, which in turn promotes PI3K/AKT signaling.

## Figures and Tables

**Figure 7:**
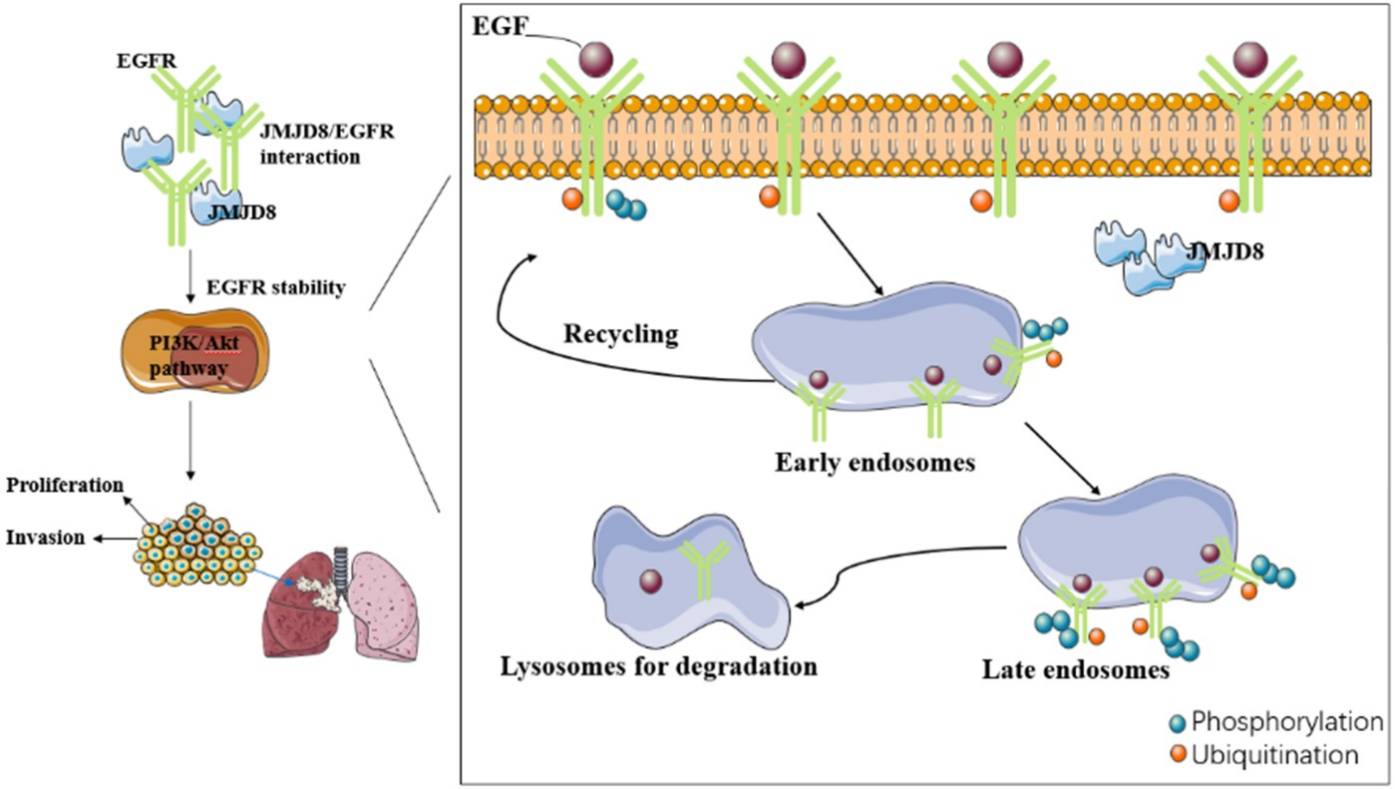
[missing figure legend]

**Table 1:** The relationship between JMJD8 expression and clinicopathological parameters of NSCLC

Clinicopathological characteristics	Total N	JMJD8-negative	JMJD8-positive	*P*-value
**Age (years)**				
≤60	89	36	53	0.550
>60	80	36	44	
**Gender**				
Male	126	48	78	0.042
Female	43	24	19	
**Histological type**				
Squamous cell carcinoma	92	53	39	<0.01
Adenocarcinoma	77	19	58	
**Differentiation**				
Well-Moderate	94	51	43	<0.01
Poor	75	21	54	
**Tumor size (cm)**				
≤3	94	33	61	0.027
>3	75	39	36	
**Lymph node metastasis**				
Negative	88	43	45	0.086
Positive	81	29	52	
**TNM stage**				
I	68	36	32	0.026
II-III	101	36	65	

NSCLC, non-small cell lung cancer.
